# Surgical Management of Patients with Chiari I Malformation

**DOI:** 10.1155/2012/640127

**Published:** 2012-06-28

**Authors:** John Siasios, Eftychia Z. Kapsalaki, Kostas N. Fountas

**Affiliations:** ^1^Department of Neurosurgery, University Hospital of Larissa, School of Medicine, University of Thessaly, Biopolis, 41110 Larissa, Greece; ^2^Department of Diagnostic Radiology, University Hospital of Larissa, School of Medicine, University of Thessaly, Larisa, Greece; ^3^Institute of Biomedical Research and Technology (BIOMED), Center for Research and Technology—Thessaly (CERETETH), 38500 Larissa, Greece

## Abstract

Chiari malformations (CMs) constitute a variety of four mainly syndromes (I, II, III, and IV), which describe the protrusion of brain tissue into the spinal canal through the foramen magnum. These malformations frequently occur in combination with other pathological entities such as myelomeningocele, hydrocephalus, and/or hydrosyringomyelia. The recent improvement of imaging techniques has increased not only the rate of CM diagnosis but also the necessity for its early treatment. Several different surgical techniques have been employed in the treatment of patients with symptomatic CM-I. In our current study, a systematic and critical review of the pertinent literature was made for identifying the most commonly employed surgical procedures in the management of these patients. Emphasis was given in outlining the advantages and disadvantages of each surgical approach. Moreover, an attempt was made for defining those parameters that may be prognostic factors for their surgical outcome. There is a consensus that surgical treatment is reserved only for symptomatic patients with CM-I. It has also been postulated that early surgically intervention is usually associated with better outcome. Despite the large number of previously published clinical series, further clinical research with large-scale studies is necessary for defining surgical treatment guidelines in these patients.

## 1. Introduction 

Chiari malformations (CMs) constitute a group of different clinicopathological entities with varying etiology, pathophysiology, and clinical features. They represent varying degrees of hindbrain herniation through the foramen magnum. Professor Hans Chiari (1851–1916) developed a four-tier scheme for classifying these entities [1, 2]. His initial description was based on the findings of more than 40 autopsies, which he had performed while he was working as a pathologist in Prague [1, 2]. 

Chiari malformation type I (CM-I), initially described in 1891, constitutes a syndrome involving the caudal descent of the cerebellar tonsils through the foramen magnum for at least 3–5 mm. It may be occasionally associated with an elongated fourth ventricle. Usually, it is a disorder of mesodermal origin, but neuroectodermal and acquired forms have also been reported [3]. Chiari malformation type II, described in 1896, consists of caudal descent of the cerebellar vermis, the fourth ventricle, and the lower brain stem and almost always is seen in conjunction with a myelomeningocele. It is the most common form and is also called the Arnold-Chiari malformation. Hydrocephalus always coexists with CM type II and may be associated with spina bifida and other cerebral, spinal, and meningeal abnormalities. The syndrome has neuroectodermal origin [3]. Chiari malformation II remains the leading cause of death in patients with treated myelomeningocele [4, 5]. 

The initial description of CM type III was based on the description of a single case with a large dermal sac in the occipital region, containing herniated cerebellum. Type III is characterized by caudal displacement of the medulla and herniation of part of the cerebellum into an occipitocervical meningocele. Sometimes, part of the hindbrain is also herniated. Hydrocephalus is present in 50% of these cases and is always of obstructive etiology, due to either aqueductal stenosis or an associated Dandy-Walker malformation. Chiari type III is a neuroectodermal malformation [3]. Type IV CM represents a pathological entity of cerebellar hypoplasia and not actual herniation. It is the least frequent form of Chiari malformations and is characterized by hypoplasia or aplasia of the cerebellar hemispheres, and morphological alterations of the pons, as well as by a pigeon-breast deformity of the brainstem. Hydrocephalus is quite rare among patients with CM-IV. It is a disorder of neuroectodermal origin [3].

During the last years, many researchers have described another form, Chiari type 0, which is characterized by an alteration in cerebrospinal fluid (CSF) hydrodynamics, at the anatomical level of the foramen magnum. It is a pathological entity characterized by some degree of posterior tilt of the pons and the medulla, with caudal displacement of the medulla oblongata, a low tip of the obex, and normal position of the cerebellar tonsils. Patients with this CM type also demonstrate syringomyelia, either without tonsil herniation or only with mild tonsil herniation [6, 7]. Mottolese et al., as many other neurosurgeons, have expressed their objections regarding the existence of this type of CM [8]. Occasionally, a caudal displacement of the brainstem, along with cerebellar tonsil ectopia, in the absence of spina bifida may be seen. This syndrome has been considered as a separate form of Chiari malformation and is called CM type 1.5 [9]. 

Pathogenesis of CMs has been extensively studied by several researchers, who have developed various explanatory theories. Examples of these theories are the developmental arrest due to myeloschisis [10–13], the overgrowth theory, which suggest enlargement of the neural plate prior to neurulation preventing thus fusion of the neural folds [14], the neuroschisis theory [15, 16], the neuroectodermal-mesodermal spatial dyssynchrony theory [12], and the traction theory [17]. In regard to the pathogenetic mechanisms implicated in the development of CM syringomyelia, which frequently coexists with CM-I, Gardner, in 1965, developed the hydrodynamic theory, which postulates that the lack of perforation of the rhombencephalic roof and the persistence of a patent communication between the fourth ventricle and the central canal of the spinal cord could lead to syrinx development [18]. Oldfield and his coworkers reported another theory for the creation of a syringomyelic cavity, based on their Cine MRI and their intraoperative ultrasonographic findings [19]. According to their theory, the Chiari malformation-associated anomalies could induce a piston-like motion, thus affecting the cerebellar tonsils and producing a systolic wave in the CSF flow, which acts on the spinal cord and induces CSF leakage through the interstitial and the perivascular spaces [19]. Nishikawa et al. studied the posterior fossa morphology in 30 sporadic cases of CM-I anomalies and 50 control cases, and they reported underdevelopment of the occipital bone along with compression of the cerebellum and the brainstem, which resulted into their caudal herniation through the foramen magnum [20]. In addition, several other authors have reported the development of an acquired CM-I, secondary to the prolapse of the cerebellar tonsils through the foramen magnum in patients with previously inserted ventriculoperitoneal or lumbar-peritoneal shunts [21, 22]. Occipital hypoplasia, posterior fossa anatomical elements' overgrowing, decreased volume of the osseous posterior fossa, underdevelopment of the occipital bone, presence of posterior fossa vascular malformations, hydrocephalus, posterior fossa masses, lumbar-peritoneal shunts, and craniofacial and posterior cranial base malformations may contribute to the development of Chiari malformations. 

The aim of our current study is to review the pertinent literature regarding surgical treatment of CM-I, with special emphasis on different surgical approaches and their modifications, as well as the identification of those factors, which may affect the outcome of surgical treatment of these patients. 

## 2. Material and Methods

An extensive literature search through the PubMed medical database was performed using any possible combination of the terms “Arnold-Chiari malformation,” “Chiari malformation,” and “Chiari I” with “decompression,” “encephalocele,” “hindbrain herniation,” “hydromyelia,” “myelomeningocele,” “occipital,” “posterior fossa,” “shunt,” “surgery,” “syringomyelia,” “syringo-peritoneal,” “syringe-pleural,” and “syringostomy.” The search was not limited by language, publication characteristics, and/or publication status. All titles and abstracts identified were meticulously reviewed. Furthermore, reference lists from the retrieved articles were carefully reviewed to identify any additional pertinent articles for inclusion. Eligible articles had English-language abstracts and were published in peer-reviewed journals. 

Every effort was made to identify any repetition of cases among the published clinical series and/or repetition of reports in different journals. In these cases, only the original clinical series were included in our study. It has to be mentioned, however, that this task was not easy and the reader must be aware of potential redundancies in the reported data. In reviewing the previously published clinical series, particular attention was paid to the design of each clinical study and its methodological characteristics, and special attention was paid on the surgical treatment and outcome of patients with CM-I. 

## 3. Results

There are several studies in the literature describing surgical techniques for treatment of Chiari type I malformation (Table 1). The basic aim of all modern surgical procedures is the restoration of normal CSF circulation at the level of the foramen magnum by performing decompression of the inferior cerebellum and of the cervicomedullary region. The restoration of CSF flow and the reestablishment of a pressure balance between the intracranial and intraspinal subarachnoidal spaces is the main goal of all employed surgical procedures.

It has to be emphasized that surgical treatment is indicated only for symptomatic patients with CM-I. The Pediatric Section of the American Association of Neurological Surgeons has clearly stated that surgical decompression has no indication as prophylactic treatment in asymptomatic children [23]. Likewise, Nash et al. in their review article support that surgical treatment is indicated only in symptomatic CM-I patients with radiographic evidence of hindbrain abnormalities [24]. Similarly, Nagib who studied 16 CM-I cases concludes that the most significant prognostic factor is the presence of clinical symptoms and signs such as scoliosis, headache, cervical pain, and/or sleep apnea [25]. Their presence indicates favorable response to surgical posterior fossa decompressive procedures [25]. However, when symptomatology is present, early surgical intervention is advocated. Dyste and Menezes suggested that symptomatic children with CM-I should undergo immediate surgery in the hope of optimizing the surgical outcome [26]. 

Numerous clinical series advocate ample posterior fossa craniectomy, including suboccipital craniectomy and removal of the C1 posterior arch, for decompressing the cerebellum and the cerebellomedullary junction, along with an augmentative duraplasty for treating symptomatic patients with CM-I. Krieger et al. reported a series of 31 children, who underwent bony decompression of the posterior fossa with dural opening but without any intradural surgical maneuvers [27]. An associated syringomyelia was present in 84% of their patients. A suboccipital craniectomy along with removal of the posterior arch of C1 and an augmentative duraplasty was performed in all their cases. They reported significant postoperative improvement of their patients' symptomatology, with persistent postoperative headache occurring in 26% and nausea in 16% of their patients. In 88% of their syringomyelia patients, a significant reduction in the size of the associated syrinx was also obtained after posterior fossa decompression. They reported that a shunt insertion was required in only three patients for their persistent syringomyelia [27]. Similarly, Zérah suggested posterior fossa decompression without intradural maneuvers, based on their experience from treating 188 children with Chiari I malformation and hydro-syringomyelia [28]. They reported 95% of clinical improvement and/or symptom arrest in their patients [28]. Lazarref and Valencia-Mayoral in their study support that treatment of Chiari malformation I with syringomyelia is surgical [29]. They suggest that posterior fossa decompression and atlas laminectomy are sufficient for treating these patients, when a ventricular shunt is present. They found in their series that if surgical intervention is performed before permanent structural damage occurs, up to 88% of these patients have significant remission of their symptoms [29]. Likewise, Yundt et al. claimed that, in children younger than 2 years old, development of arachnoidal adhesion is extremely rare; therefore, lysis of arachnoidal adhesion does not alter their surgical outcome [30].

The role of duraplasty performance in treating patients with CM-I has been questioned by many clinical investigators. Hankinson et al. in their review study compared posterior fossa decompression without duraplasty to posterior fossa decompression with an expandable duraplasty [41]. They concluded that hindbrain decompression through suboccipital craniectomy and removal of the posterior arch of the atlas is the current first line of treatment for patients suffering CM-I. The authors emphasized, however, that the wide clinical and pathophysiological spectrum of children with CM-I allows no general recommendations for the surgical treatment and the most suitable approach for these patients [41]. 

Isu et al. suggested foramen magnum decompression along with removal of only the outer dural layer for treating syringomyelia associated with CM-I [31]. They presented a series of 7 children and adolescents with hydro-syringomyelia, who had undergone previous insertion of syringosubarachnoid shunts. They reported that 88% (6/7) of their patients demonstrated improvement of their preoperative symptomatology after undergoing foramen magnum decompression and outer dural layer reconstruction. Furthermore, in 5/7 of their patients (71.4%) there was immediate postoperative decrease of the syrinx, a finding that was progressively observed in all their patients [31]. Genitori et al. studied the role of posterior fossa bony decompression in the management of symptomatic children affected by CM-I [32]. They included in their study 53 patients, who were divided into asymptomatic patients, who received no surgical treatment and were only subject to clinical observation, and symptomatic patients (brainstem compression in 16, syringomyelia in 10, including 7 holocord syrinx cases). All the symptomatic patients were treated with the same surgical approach: bony decompression of posterior fossa with suboccipital craniectomy along with removal of the posterior arch of C1 and removal of the outer layer of the dura without actual arachnoidal opening. In all symptomatic patients with brainstem compression, there was resolution of their preoperative symptomatology. In patients with syringomyelia, their syrinx-associated symptoms were resolved or improved in 94.4% of the cases. Their overall improvement rate was 97.2%. This series seems to demonstrate that even a simple extradural surgical approach, with a lower rate of postoperative complications and shorter stay in the hospital, is sufficient to arrest the progress of the disease and to improve the preoperative symptomatology in a high percentage of cases [32].

 Their results were comparable to those achieved with other, more aggressive surgical procedures including extensive duraplasty, arachnoidal adhesion lysis, and resection of the cerebellar tonsils. Alden et al. in their retrospective study examined 21 patients (11 patients with isolated CM-I and 10 patients with CM-I and syringomyelia) undergoing suboccipital craniectomy, C1 posterior arch removal, and duraplasty, including arachnoidal adhesion lysis, and cerebellar tonsil resection [33]. In four of their patients durotomy and duraplasty were performed with no further intradural exploration. In the rest of their patients, intradural exploration with resection of the cerebellar tonsils and lysis of the arachnoidal adhesions and an augmentative duraplasty was performed. Symptoms resolved in 14 patients (67%), significantly improved in 6 (29%), while remained the same in 1 patient (4%). In 10 of their patients with syringomyelia, the syrinx resolved in 8, decreased in one, and remained the same in another patient [33].

The usage of dural graft and the characteristics of the graft material may influence the surgical outcome in patients undergoing surgery for CM-I, since duraplasty is widely performed. Parker et al. in their retrospective study hypothesized that a recently observed increase in complications of posterior fossa decompression was caused by the utilized graft [34]. They claimed that complication rates after CM-I decompression may be dependent on the dural graft and the usage or not of tissue sealant. The complication rate at the authors' institution approximately doubled following the adoption of a different graft product. Characteristically, their complication rate before the usage of dural allografts was 18.1% and with the allograft increased to 35%. Tissue sealants used in combination with a dural substitute to augment the strength of the duraplasty may increase the risk of aseptic meningitis and/or postoperative CSF leak [34]. Similarly, Mottolese et al. in their comparative study reported their results from two groups of patients treated with the same surgical procedure (suboccipital craniectomy, C1 posterior arch resection, and augmentative duraplasty) [8]. The only difference between these two groups was the usage of Gotorex dural patch, which seemed to be associated with better surgical outcome. The observed symptom remission rate was 70% for the group without Gotorex, while the respective percentage was 89% for the Gotorex group. However, the reported complication rate was 18% among patients with no Gotorex, while the respective percentage for the Gotorex group was 20.5% [8]. 

 Furthermore, Valentini et al. reported a series of 99 children who were operated for CM-I alone or with syringomyelia [35]. They performed suboccipital craniectomy with posterior arch removal of the atlas and duraplasty in one group of their patients, while suboccipital craniectomy with atlas posterior arch removal, duraplasty, and cerebellar tonsil coagulation/resection were performed in another group. Their cumulative symptom improvement rate was 78%, while syrinx decrease was observed postoperatively in 91.5% of their patients. They reported 14% postoperative CSF leakage, for which a reoperation was required. The authors found that preoperative symptoms improved more in their pediatric than in their adult cases, treated at the same institution during the same period. Their explanation was the shorter symptom duration and the increased plasticity of the pediatric nervous system. The results of the limited osseous extradural decompression were poor in their series. The authors concluded that the clinical symptoms are often more serious in children than in adults, but the results of surgery are better in the pediatric population [35]. 

The extent of the posterior fossa craniectomy, as well as the total surface of the duraplasty, represents two other controversial issues in the surgical management of patients with CM-I. Sindou et al. suggest a procedure, which consists of a suboccipital craniectomy and a C1 (and occasionally C1 and C2) laminectomy, plus an extreme lateral foramen magnum opening, along with a Y-shaped dural incision with preservation of the arachnoid membrane, and an expandable duraplasty [36, 42]. Their series included 44 patients with symptomatic CM-I. Fifteen patients had CM-I with associated syringomyelia (34%), while 29 patients (66%) had CM-I with no syringomyelia. Preoperatively, 37 patients (84.1%) were functionally independent (of whom 13 had syringomyelia), while 7 patients (15.9%) were functionally dependent (two of these with syringomyelia). There was no operative mortality, and surgery did not elicit any new neurological deficits. Postoperatively, all their patients were functionally independent. For those patients with isolated CM-I, the average postoperative Karnofsky performance score was 90 versus 76 before surgery. For those patients with CM-I and syringomyelia, the average postoperative Karnofsky score was 89 versus 74 before surgery. Additionally, they reported that syrinx size was postoperatively decreased in 60%, while in 40% it remained stable with no further increase. Their postoperative CSF leakage was 4.5%, while their surgical wound infection rate was 11.4%. They concluded that this type of craniocervical decompression achieved the best results with minimal complications and side effects [36, 42]. 

The surgical treatment of the Chiari-associated syringomyelia and the selection of the most efficient surgical strategy for this represent another puzzling therapeutic dilemma. Hoffman et al. reported a series of 47 cases of hydro-syringomyelia including 12 patients with CM-I and 5 patients with acquired CM-I [37]. The authors performed in 31 of their patients a Gardner's procedure (suboccipital craniectomy, C1 posterior arch removal, obex plugging, and augmentative duraplasty), while 5 patients underwent posterior fossa decompression and duraplasty, 9 patients had a shunt insertion alone, and 2 patients a posterior fossa decompression and duraplasty along with shunt insertion. Their overall success rate was reported to be 70%, with improvement of the preoperative symptomatology of their patients. They stated that posterior fossa decompression with dural grafting alone was not enough for relieving patients' symptomatology [37]. 

Hida et al. compared the results of two major surgical procedures, posterior fossa along with C1 decompression versus syringosubarachnoid (SS) shunting, for treating patients with CM-I and syringomyelia [38]. They included 70 patients in their series (age range: 3–59, median age: 29.4 years). Posterior fossa and C1 decompression were performed on 33 patients, and SS shunting was performed on 37 patients. They principally performed posterior fossa and C1 decompression in patients with symptoms of CM-I without or with a small syrinx. They preferred to employ SS shunting only in patients with large syringes. Postoperative magnetic resonance imaging demonstrated that the syrinx had totally collapsed or decreased in size in 94% of the patients who underwent posterior fossa along with C1 decompression and in 100% of the patients undergoing SS shunting. Neurological improvement was observed in 82% and in 97% undergoing posterior fossa and C1 decompression and SS shunting, respectively. The average time for the syrinx to collapse was 6.3 weeks after surgery in the posterior fossa and C1 decompression group and 1.8 weeks in the SS shunting group. Their results indicate that clinical symptoms and radiological findings improved faster in the SS shunting group than in the posterior fossa and C1 decompression group [38].

 Surgical management of CM-I has as a result correction of the frequently associated kyphosis/scoliosis. Eule et al. reported their experience from a retrospective review of 25 patients (age range: 1.5–16.5 years) with CM-I and kypho- scoliosis [39]. Scoliotic curves were classified by magnitude and curve type. All patients were treated with surgical decompression for the CM-I with or without drainage of the associated syringomyelia. Nineteen patients (76%) had associated scoliosis. The majority of the patients with scoliosis (13 of 19) sought treatment for their spinal deformity and only 6 for pain and/or neurological symptoms. Eleven of 19 patients with scoliosis (58%) underwent spinal fusion. Eight of 19 (42%) patients have not undergone fusion: 3 had experienced progress, 1 remained in a stable condition, and 4 had experienced improvement of curvature after their posterior fossa decompression. The mean age of patients who experienced progress after decompression was 14.5 years, compared to 6 years for patients who experienced improvement. The authors concluded that early decompression of Chiari malformation type I with syringomyelia and scoliosis resulted in improvement or stabilization of the spinal deformity in 5 cases. Each of these patients underwent decompression before 8 years of age and before the scoliotic curve becomes severe [39]. 

Surgical treatment may be a valid treatment option even for acquired forms of CM-I. Payner et al. reported the cases of 10 children with lumbar-peritoneal shunts in whom previous radiographic studies had confirmed a normal hindbrain configuration [40]. Seven of the 10 patients demonstrated de novo tonsillar descent into the foramen magnum, detected by magnetic resonance imaging, whereas the others remained normal. Four of seven patients were symptomatic: two underwent removal of the lumbar-peritoneal shunt and conversion to a ventriculoperitoneal shunt and two underwent posterior fossa decompression. Further postoperative magnetic resonance imaging revealed that one of the two patients who underwent conversion showed ascent of the cerebellar tonsils. All four patients became asymptomatic in less than 6 months after surgery. The authors concluded that a craniospinal pressure gradient creates a potential for downward displacement of the cerebellar tonsils; therefore, they recommended that ventriculoperitoneal shunting is preferable in children with communicating hydrocephalus, in order to avoid this potential complication. They also recommended annual imaging of the cervicomedullary junction in children with lumbar-peritoneal shunting. Finally, if symptomatic tonsillar herniation occurs due to lumbar-peritoneal shunting, a trial conversion to ventriculoperitoneal shunting could eliminate the need for posterior fossa decompression [40]. 

The wide spectrum of CM-I-associated symptomatology, along with the significantly varying regional anatomy, makes the accurate interpretation of the results of each clinical series extremely difficult and the comparison of outcome rates among different series almost impossible. Tubbs et al. in their review study examining the surgical outcome in pediatric patients with CM-I concluded that there is significant variation in the reported anatomy, surgical treatment, and outcome in the published clinical series [43]. Likewise, Fernández et al. in their study for malformations of the craniocervical junction concluded that there is no consensus among the specialists regarding the etiology of CM-I or how to approach, surgically treat, and follow, these patients [44]. Similarly, Oró and Mueller in their review study noted that there is no consensus on the procedural steps that lead to a consistently favorable outcome in patients with CM-I [45]. 

## 4. Discussion

The management of patients with Chiari malformation type I remains a puzzling issue, despite the better understanding of the involved pathophysiological mechanisms and all the recent advances in imaging. There is a general consensus that asymptomatic patients with CM-I are not generally considered candidates for surgical treatment [23]. A previous large-scale survey performed among board-certified pediatric neurosurgeons for the pediatric section of the American Association of Neurological Surgeons has demonstrated that there is a general agreement that surgery should not be carried out on asymptomatic patients with CM-I [23]. The same study concluded that the presence of brainstem dysfunction, cranial nerve dysfunction, hydro-syringomyelia, and kyphosis associated with CM-I are indications for surgical intervention [23]. Moreover, a recently published study by Strahle et al. examined the natural history of patients diagnosed with CM-I [46]. The authors postulated that the natural history in the vast majority of patients with CM-I is benign, and in their long-term follow-up study there was no change in the amount of tonsillar herniation or in the CSF circulation pattern [46].

 In those cases, however, symptomatology indicates surgical intervention, there is a growing body of evidence supporting early surgical intervention [25, 26, 28, 29, 39]. Furthermore, even when surgical management is clearly indicated, there are various surgical approaches and numerous modifications available for the treatment of these patients [26–28, 30–33, 36, 38, 41, 42]. The presence of several surgical procedures for treating CM-I is exactly indicating that none of these procedures works perfectly for all patients. The vast majority of the previously published clinical series indicate that suboccipital craniectomy, removal of the posterior arch of C1, and an augmentative duraplasty represent a baseline surgical approach, which is applicable in most Chiari I patients and is performed by the majority of the involved neurosurgeons. In CM-I, suboccipital posterior fossa decompression and atlas laminectomy are considered the standard surgical approach to the vast majority of symptomatic patients [24, 27, 28, 32, 41]. Several clinical series have reported 95%–97% of improvement in preoperative symptomatology of their patients [24, 27, 28, 32, 41]. On the other hand, good or excellent outcome has also been reported from employing alternative surgical procedures with modified osseous posterior fossa decompression [33, 34, 36, 42]. The performance of an extensive suboccipital craniectomy and the far lateral opening of the foramen magnum proposed by Sindou et al. [36, 42] are not proven to be superior to the standard-size suboccipital craniectomy. In addition, the extended craniectomy may predispose to increased vascular injury operative risk during the lateral extension, prolonged operative time, and increased incidence of postoperative CSF leakage.

Similarly, the performance of extensive arachnoidal adhesion lysis has not demonstrated to be superior to performing duraplasty alone. It has to be emphasized, that in cases of reoperation, the employment of arachnoid dissection may be advantageous for opening adhesions and thus restoring CSF circulation. Furthermore, the identification of arachnoidal veils or compartmentalization of the posterior fossa on the preoperative MRI is an indication for extensive arachnoidal dissection, in order to restore the compromised CSF circulation [43]. However, in first-time cases, and especially in children, the presence of arachnoidal adhesions is extremely rare. Besides, the arachnoidal dissection itself may be a strong stimulus for further postoperative arachnoidal adhesion development. Likewise, the shrinkage or the resection of the cerebellar tonsils represents another controversial point. Unfortunately, there are no comparative studies available for evaluating the exact role of this surgical maneuver. In those cases that cerebellar tonsils are highly gliotic or massive, their resection may help in restoring the CSF circulation. It has to be mentioned, however, that particular attention has to be paid to the adjacent posterior inferior cerebellar arteries during tonsil resection or shrinkage. Additionally, it has to be kept in mind that cerebellar tonsil resection may cause increased incidence of postoperative nausea and vomiting. The results of Alden et al. [33] and Valentini et al. [35], who employed in their reported series tonsil resection, were similar to those of other series with no tonsillar resection. The obstruction of CSF flow through the obex is another surgical technique, which has been reported for being employed during the surgical management of patients with CM-I [37]. It has not been demonstrated, however, that its application improves the surgical outcome of these patients.

The performance of an expandable and augmentative duraplasty for avoiding any postoperative compression of the posterior fossa contents seems to be widely accepted [31, 33, 34, 36, 42]. However, there are anecdotal reports for performing no duraplasty in cases of children with CM-I. Durham and Fjeld-Olenec in their meta-analysis study compared the results of posterior fossa decompression with and without duraplasty [47]. They found that the two techniques have similar results in regard to the postoperative clinical improvement and the decrease of the associated syrinx. They noticed that duraplasty is associated with a lower risk of reoperation but carries a greater risk for postoperative CSF leakage [47]. Moreover, the material used for performing the duraplasty constitutes another point of controversy. It has been postulated that the usage of autograft or allograft and the type of the allograft may influence the postoperative complication rate and the patient's overall surgical outcome. Mottolese et al. [8] reported that the usage of Gotorex along with a tissue sealant resulted in better outcome rates in their series. However, the authors noticed an increased incidence of complications in those cases that a dural allograft and a tissue sealant were utilized [8]. The possibility of developing aseptic meningitis in cases of dural allografts and/or tissue sealants should be addressed in a prospective clinical study.

The association of CM-I and syringomyelia is a well-established one. However, the treatment strategy for syringomyelia in these patients remains still controversial. The indications for surgical intervention were ill-defined until recently, when a large-scale survey was performed for the American Society of Pediatric Neurosurgeons, examining the current treatment trends of patients with CM-I and syringomyelia [48]. The survey showed that the vast majority of pediatric neurosurgeons in the USA reserve surgical treatment only for symptomatic patients, while they prefer to follow asymptomatic cases with clinical and imaging evaluations. In those cases that surgical intervention is decided, there is no general consensus in regard to the type of the employed surgical procedure, although the majority of the participants favor posterior fossa decompression as the initial surgical approach. It is also reported in the same study that syrinx drainage has been abandoned as a surgical treatment, in the vast majority of cases [48]. There are several clinical series demonstrating that arresting of the progression of CM-I after posterior fossa surgical decompression may also improve the patient's symptoms related to the underlying syringomyelia [31–33, 36, 42]. Several investigators support the idea that posterior fossa decompression seems to be more effective than syrinx shunting procedures in patients with CM-I [31–33, 36, 42]. It has to be noted, however, that there are also many reports advocating the employment of a syringotomy or syringo-peritoneal shunting for managing the CM-I-associated syringomyelia [25, 31, 36, 38, 42]. This latter approach may be indicated as a first-line surgical treatment in cases of extensive, large syringomyelic cavities. It has also been demonstrated that syrinx shunting results in faster decrease in the syrinx size compared to posterior fossa decompression [38]. A multicenter trial of surgical outcome in patients with CM-I and syringomyelia is underway for accurately evaluating the outcome and the complication rates of each surgical methodology and for outlining the most efficient surgical approach. 

Selection of the most suitable time for surgical intervention in patients with mild symptomatology secondary to CM-I raises another puzzling question for clinicians. It has been reported by Dyste and Menezes that the timing of surgical intervention in children suffering CM-I plays an important role in the outcome of these patients [26]. They suggested immediate surgery in order to improve surgical outcome [26]. Likewise, Valentini et al. reported that surgical outcome was better in their pediatric compared to their adult cases [35]. They believe that the shorter duration of preoperative symptomatology was of paramount importance in the improved outcome of their pediatric patients [35]. It has also been postulated by Eule et al. that early surgical correction of CM-I improves the outcome of the associated kyphosis/scoliosis in these patients [39].

Surgical intervention in cases of CM-I has been associated with a wide spectrum of intraoperative and postoperative complications. Meticulous knowledge of all potential complications is of paramount importance for their complete prevention or their minimalization and also for their early and prompt management when they occur. Thorough knowledge of the posterior fossa anatomy is mandatory for avoiding any vascular injuries to a remnant circular venous sinus (in cases of young children), the extracranial vertebral arteries, or the posterior inferior cerebellar arteries. Meticulous intraoperative coagulation is important for avoiding significant blood loss and also for preventing the formation of any postoperative hematomas. Prompt replacement of any blood loss is critical particularly in pediatric cases. The postoperative development of a CSF leakage is the most common complication in these patients, independent of the type of the employed surgical approach. Meticulous water-tight dural closure is important for avoiding any postoperative leaks. The role of tissue sealants in preventing postoperative CSF leaks remains to be defined in prospective comparative studies. The benefit from their usage, if any, has to be counterweighted, however, with the risk of developing postoperative aseptic meningitis. Surgical wound infection represents another occasionally troublesome complication, which may be limited by employing strict sterilization techniques, by administering the appropriate perioperative antibiotic prophylaxis, and by minimizing the operative time. The exact role of dural allografts, tissue sealants, and other implants, in the development of surgical infections in cases of CM-I, remains to be addressed. Finally, early postoperative ambulation of the patients may help preventing any other procedure-related general complications, which are generally quite rare in patients with CM-I, mainly due to their young ages. More rare complications such as postoperative development of acute hydrocephalus or acute brainstem compression have also been reported [43]. Tubbs et al. [43] reported a cumulative complication rate of 2.3% in their series, while Mottolese et al. [8] reported complication rate ranging between 18 and 20.5% in their cohort. 

It is apparent that there is a significant variation in the employed surgical strategies in the management of patients with CM-I. Tubbs et al. [43], Oró and Mueller [45], and Fernández et al. [44] concluded that there is a significant variation in the reported anatomy, outcome, and surgical treatment options for children with CM-I. Similarly, Eule et al. concluded that there is no consensus on the procedural steps, which lead to a consistently favorable outcome in CM-I patients [39]. Indeed, there is no consensus among the specialists regarding the etiology of the disease or how to approach, treat, and follow up the CM-I cases. It is necessary that physicians involved in the care of people with this condition should comprehensively approach the management and the follow up of their patients. Interdisciplinary teams should be organized including all the professionals, who can help to increase the quality of life of these patients. The individualization of treatment in patients with CM-I cannot be overemphasized. However, standardized treatment paradigms based on randomized controlled studies are still necessary to outline strict surgical treatment criteria and to identify the most suitable surgical approach for each patient. 

## 5. Conclusions

It is generally accepted that Chiari malformations constitute a well-defined clinicopathological entity. There is, however, no consensus regarding the classification of different types of the Chiari malformations, while significant variation exists in the surgical management of these patients. It is widely accepted that in CM-I cases that surgical treatment is advocated only in symptomatic patients, since the natural history of the disease seems to be quite benign in asymptomatic cases. There is a trend in the literature indicating that the earlier the surgical intervention is, the better the functional outcome of a patient will be. There are several different surgical approaches for these patients indicating exactly that none of them can be universally employed. The surgical treatment should be individualized for each patient. Suboccipital craniectomy along with atlas' posterior arch removal and augmentative duraplasty seems to be the most commonly performed procedure. The exact clinical importance of arachnoidal dissection, coagulation and shrinkage/resection of the cerebellar tonsils, and/or obex plugging remains to be defined. Similarly, the most suitable surgical approach for patients with CM-I and syringomyelia remains to be defined, although the majority of pediatric neurosurgeons seem to favor posterior fossa decompression as first-line treatment in symptomatic syringomyelia patients. Large-scale, prospective studies have already been initiated and are underway for addressing all these controversial issues.

## Figures and Tables

**Table 1:** Synopsis of previously published clinical series including the number of their patients, the underlying pathologies, the employed surgical approach, and the outcome and complication rates.

Authors year	Pts. number	Pathology	Surgical approach	Outcome	Morbidity Mortality Complications
Krieger et al. [27], 1999	31	CM-I, CM-I + hydro-syringomyelia	Occipital craniectomy, C1 posterior arch removal, and duraplasty	88% improvement in pts. with syrinx 3 patients required also a shunt	26% headaches 16% nausea No mortality

Zérah [28], 1999	188	CM-I + hydro-syringomyelia	Posterior fossa decompression	95% improvement/ stabilization of symptoms	N/A

Lazarref and Valencia-Mayoral [29], 1990	N/A	CM-I + hydro-syringomyelia	PFD + cervical laminectomy if the ventricular shunt is patent	88% symptom remission	N/A

Yundt et al. [30], 1996	3	CM-I	Occipital craniectomy, C1 posterior arch removal, and duraplasty	100% improvement	N/A

Isu et al. [31], 1993	7	CM-I + hydro-syringomyelia	Occipital craniectomy and duraplasty	6/7 improvement, 5/7 immediate syrinx decrease	N/A

Genitori et al. [32], 2000	53	CM-I, CM-I + hydro-syringomyelia	Occipital craniectomy, C1 posterior arch removal, and duraplasty	100% improvement in brainstem compression, 94.4% syringomyelia improvement, 97.2% overall improvement	2/34 pts. required second surgery. No mortality

Alden et al. [33], 2001	21	CM-I, CM-I + hydro-syringomyelia	suboccipital craniectomy + cervical laminectomy in all pts (i) durotomy + duraplasty: 4 (ii) resection of cerebellar tonsils + lysis of abhesions + duraplasty: 17	67% symptom resolution, 29% improvement, 4% no improvement	N/A

Parker et al. [34], 2011	114	CM-I	Occipital craniectomy, C1 posterior arch removal, and duraplasty with or without tissue sealant (15 cadaveric pericardium-12 Durepair-87 endura)	N/A	Graft-type and Cx rates: 26,7% cadaveric pericardium: 26.7% durepair: 41.7 % endura: 17.2% Reoperation rates: cadaveric pericardium: 13% durepair: 25% endura: 8.1% Complications rates for tissue sealants: no sealant: 14,8% tisseel: 18,7% duraseal: 50% Combined complication rate for durepair and DuraSeal: 56% Cumulative Cx rate: 21.1%

Mottolese et al. [8], 2011	82	CM-I, CM-I + hydro-syringomyelia, kyphosis/scoliosis	(i) Occipital craniectomy, C1 posterior arch removal, and duraplasty (Group a: 43 pts) (ii) Occipital craniectomy, C1 posterior arch removal, and duraplasty with Gore-Tex dural patch (Group b: 39 pts)	Group a: 70% improvement Group b: 89% improvement	Group a: 18% complication rate Group b: 20,5% complication rate. No mortality

Valentini et al. [35], 2011	99	CM-I, CM-I + craniosynostosis, CM-I + hydrosyringomyelia	Craniovertebral decompression (Group a: 7 pts), craniovertebral decompression with duraplasty combined with tonsillar coagulation (Group b: 44 pts)	91.5% syrinx decrease, 78% overall symptom improvement	No mortality 14% reoperation for CSF leakage, 6.8% oculomotor n. dysfunction, 2.8% venous thrombosis

Sindou et al. [36], 2002	44	CM-I, CM-I + hydro-syringomyelia	Craniocervical decompression + far lateral foramen magnum opening + duraplasty with arachnoid preservation.	Improvement based on KPS, improvement of syrinx in 60%, stabilization of syrinx in 40%	4.5% CSF leakage, 2.3% laryngeal edema, 2.3% pneumonia, 11.4% wound infections No mortality

Hoffman et al. [37], 1987	47	CM-I, acquired CM-I	31 pts posterior fossa decompression and plugging of the obex, 5 pts posterior fossa decompression, 9 pts shunting, 2 pts decompression with shunting	70% improvement in pts undergoing obex plugging	No mortality

Hida et al. [38], 1995	70	CM-I, CM-I + hydro-syringomyelia	33 pts foramen magnum decompression (Group a), 37 pts shunting (Group b)	Group a: 94% reduced size of syrinx, 82% improvement Group b: 100% syrinx reduction, 97% improvement	N/A

Eule et al. [39], 2002	25	CM-I, CM-I + Kyphosis/Scoliosis	Decompression with or without shunt	Early decompression resulted in improvement or stabilization of scoliosis in 5 cases	N/A

Payner et al. [40], 1994	10	Acquired CM-I	2 pts conversion to ventriculoperitoneal shunt, 2 pts posterior fossa decompression	100% symptom improvement	N/A
